# Comparative Evaluation of AI-Assisted and Manual CBCT-Derived Graft Volume Measurements for Maxillary Sinus Floor Augmentation

**DOI:** 10.3390/diagnostics16142268

**Published:** 2026-07-20

**Authors:** Badr Othman

**Affiliations:** Department of Periodontics, Faculty of Dentistry, King Abdulaziz University, Jeddah 21589, Saudi Arabia; bothman@kau.edu.sa

**Keywords:** artificial intelligence, cone-beam computed tomography, dental implants, maxillary sinus floor augmentation, maxillary sinus, reproducibility of results

## Abstract

**Background/Objectives**: Accurate estimation of augmentation volume is essential for successful maxillary sinus augmentation planning. Manual CBCT-derived volumetric calculations remain time-consuming and operator-dependent. Artificial intelligence (AI)-assisted volumetric estimation may provide a standardized and reproducible alternative. This study evaluated the agreement and reliability of an AI-assisted volumetric estimation tool compared with CBCT-derived manually calculated augmentation volumes for sinus augmentation planning. **Methods**: A retrospective comparative study was conducted on 60 CBCT scans obtained from patients undergoing implant treatment planning. Radiographic measurements included bone width (A), residual alveolar bone height (B), sinus lift height (C), sinus lift width (D), and sinus lift depth (E). Manual augmentation volume was calculated using a geometric ellipsoid approximation formula derived from standardized linear measurements. AI-assisted volumetric estimation was performed using the volumetric analysis tool integrated within Planmeca Romexis 6.3 software. Two calibrated periodontists repeated both manual and AI-assisted measurements twice with a two-week interval. Reliability was assessed using intraclass correlation coefficients (ICC), while agreement between methods was evaluated using ICC and Bland–Altman analysis. **Results**: The mean manually calculated planned augmentation volume per implant site was 0.47 ± 0.11 cm^3^, whereas the mean AI-assisted planned augmentation volume was 0.52 ± 0.10 cm^3^. AI-assisted measurements were significantly greater than manual measurements (*p* < 0.001). Manual measurements demonstrated moderate intra- and inter-examiner reliability (ICC = 0.722, 0.701, and 0.649), whereas AI-assisted measurements demonstrated excellent reliability (ICC = 0.917, 0.924, and 0.903). Agreement between AI-assisted and manual volumetric estimation was good (ICC = 0.847). Bland–Altman analysis demonstrated a mean bias of 0.061 cm^3^ with limits of agreement ranging from 0.028 to 0.094 cm^3^. No significant associations were observed between augmentation volume and age, sex, or number of missing teeth after normalization per implant site (*p* > 0.05). **Conclusions**: AI-assisted volumetric estimation demonstrated excellent reproducibility and good agreement with manually calculated augmentation volumes while producing slightly higher volume estimates. AI-assisted volumetric estimation may serve as a reliable adjunctive tool for sinus augmentation planning by improving standardization and reducing operator-dependent variability.

## 1. Introduction

The lateral window and crestal techniques for maxillary sinus floor augmentation have been established as useful methods for increasing residual bone height. The maxilla generally has less vertical bone height due to resorption of the alveolar crest and subsequent pneumatization of the sinus following tooth loss, which makes augmentation of the sinus floor a cornerstone in implant dentistry [1]. Furthermore, sinus floor elevation (SFE) had been defined as a reliable surgical standard to restore volume in the atrophic posterior maxilla using radiographic analysis prior and post-operatively. Cone beam computed tomography (CBCT) was used as the imaging technique of choice because it provided an accurate 3D assessment to allow diagnosis, planning and postoperative evaluation. It allowed identification of detailed visualization of the surgical site, pathologies, prediction and virtual planning for successful procedures using minimal invasiveness with much lower morbidity to patients. Use is only standardised and justified according to diagnostic guidelines, and further exploration of the use of AI for automation of SFE management and clinical decision-making should be pursued [2]. Assessment of augmentation height and angulation allows clinicians to estimate the bone volume required for sinus augmentation [3]. After maxillary sinus floor augmentation, a decrease in the augmented volume will be unavoidable no matter which grafting material is used. In a previous study, adding xenograft to autogenous bone graft allowed to increase volumetric stability compared to autogenous bone alone. However, these conclusions should be made with caution as past work was based on three-dimensional radiographic evaluations in only four studies. Three-dimensional imaging by CBCT allows clinicians to analyze residual bone height, dimensions of the sinus cavity and graft material volume with a better accuracy compared to two-dimensional radiography [4]. Maxillary sinus volume (MSV) is greatly variable between individuals, and it is affected by changes in craniofacial morphology such as skeletal class and demographic variables such as sex and age. Statistical analysis revealed that maxillary sinus dimensions were not significantly different between right and left sides (*p* > 0.05); therefore the average of bilateral measurements was used for subsequent analyses [5]. Subsequent studies demonstrate that males generally have larger sinuses than females, which may contribute to the clinical heterogeneity of graft size needs [6]. In edentulous sites, a reduced residual ridge height and an expansion of the sinus floor was found by use of CBCT [7]. The crestal technique had provided a less invasive option for cases with residual bone height < 4 mm [8]. Seventeen Meta-analyses comparing lateral versus crestal SFA approaches have demonstrated similar implants survival if proper planning and anatomy assessment are performed [9]. Systematic reviews have shown that SFA, both grafted and non-grafted with ostetome can exhibit long-term success when properly volumetrically planned [10]. Unlike fully autonomous segmentation models, the present study evaluated a semi-automated operator-guided AI-assisted volumetric estimation tool embedded in commercially available CBCT planning Planmeca Romexis 6.3 software. Manual volumetric estimation derived from CBCT scans remains time-consuming and operator-dependent, with potential variability between examiners. Artificial intelligence (AI)-assisted volumetric estimation offers the potential to streamline preoperative planning by providing rapid, standardized, and reproducible augmentation volume calculations. Although AI-based segmentation and volumetric assessment have shown promising results in maxillofacial imaging, evidence regarding the agreement and reproducibility of commercially available AI-assisted volumetric estimation tools for sinus augmentation planning remains limited. AI–based segmentation has recently been introduced as a game-changing technique to facilitate and standardize sinus volume measurements [11]. A deep learning framework implemented with a CNN model showed automatic maxillary sinus segmentation on cone-beam compute, provided quick and accurate reproducible automatic segmentation of the MS allowing for accurate 3D model reconstruction powerful enough for diagnostic purposes and virtual treatment planning. The proposed U-Net model is capable of quickly and accurately segmenting the maxillary sinus, reducing clinician workload and potentially limiting subjective error [12]. Recent advances even combine segmentation models and pathology detection to give a complete pre-operative analysis [13]. These algorithms were subsequently validated to be reproducible across imaging systems and thereby confirms their robustness in real-world dental practice [14]. Both clear and hazy maxillary sinuses were well–segmented by a proposed deep learning model with post-processing. This may provide a clinical tool that is generalizable to many applications in dentistry with consistent accuracy [15]. Similar deep-learning systems based on craniofacial and jaw substructure segmentation have been developed with a minimum submillimetre accuracy, thus demonstrating their feasibility for potential clinical application focusing on volumetric maxillofacial assessment [16]. The accuracy of estimating graft material pre-operatively is critical to achieving the desired height and stability post operatively [17]. Manual and automated volumetric measurement frameworks evolve; threshold selection and boundary definitions exercise strong control over calculated values [18]. Comparative systematic reviews of sinus grafting with and without grafts show that precise quantification of residual bone height and volume of research remains one of the most popular aspects in predicting surgical success [19]. This indicates a strong potential for AI to automate maxillary sinus imaging and further improve the diagnostic and treatment planning quality in implantology, albeit the generalizability of these findings was moderate and could be improved by using larger and more heterogeneous datasets. Future research must focus on increasing sources of data, model interpretability and standardized AI transparency protocols [20].

Therefore, the aim of the present study was to evaluate the agreement and reliability of a semi-automated AI-assisted volumetric estimation tool compared with CBCT-derived manually calculated augmentation volumes for maxillary sinus augmentation planning. Specifically, the study assessed intra- and inter-examiner reproducibility of both manual and AI-assisted measurements, agreement between AI-assisted and manually calculated augmentation volumes, and the influence of selected demographic and clinical variables, including age, sex, number of missing teeth, surgical approach, and guided bone regeneration (GBR) requirements, on planned augmentation volume.

## 2. Materials and Methods

- Study Design

This retrospective comparative study evaluated the agreement and reliability of AI-derived planned augmentation volumetry versus manually calculated CBCT-derived sinus volume measurements in sinus augmentation planning using Planmeca ProMax 3D Mid unit (Planmeca, Helsinki, Finland) and Planmeca Romexis 6.3 software. Each sinus was analyzed twice, first using AI-assisted volumetry and then through standardized manual linear measurements by calibrated Periodontists examiners. It was reviewed and approved by the Research Ethics Committee (REC), Faculty of Dentistry, King Abdulaziz University, Jeddah, Saudi Arabia (Proposal No. 149-10-25; Approval Date: 1 December 2025). The study was conducted in accordance with the ethical principles of the Declaration of Helsinki and its subsequent amendments.

- Sample Selection

A retrospective convenience sample was used. Consecutive CBCT scans obtained from patients referred for implant treatment planning or sinus augmentation assessment between January and April 2026 at Tam Dental Clinic, Jeddah, Saudi Arabia, were screened according to predefined inclusion and exclusion criteria.

- Inclusion criteria:
  - Adults (>18 years) undergoing CBCT for implant planning.
  - CBCT imaging from patients undergoing implant planning or sinus augmentation evaluation.
  - CBCT scans with a complete maxillary sinus visible within the field of view.
  - Sinus cavity intact, no prior surgery.
- Exclusion criteria:
  - Scans with motion artifacts or metallic scatter obscuring the sinus.
  - Pathological lesions (e.g., cysts, tumors, polyps) or previous sinus grafts.
  - Incomplete visualization of the sinus cavity.

A total of 85 patients were screened, 25 were excluded and 60 patients were included. The excluded CBCT scans did not satisfy the predefined eligibility criteria because of one or more of the following reasons: motion artifacts or metallic scatter that compromised image quality, pathological lesions or previous sinus grafting, and incomplete visualization of the maxillary sinus within the field of view. The study was designed as an exploratory retrospective validation study; therefore, all eligible CBCT scans available during the study period were included.

**Demographic and Clinical Variables**

Age and sex were registered for all included subjects as demographic variables. The number of missing posterior maxillary teeth at the sinus augmentation region was also documented and categorized into, single missing tooth, two missing teeth, and three missing teeth. Clinical management decision variables were also registered, including sinus augmentation technique and guided bone regeneration (GBR) usage.

- Imaging Protocol

All scans were acquired using the Planmeca ProMax 3D Mid unit (Planmeca, Helsinki, Finland) with standardized exposure parameters (voxel size 0.2–0.3 mm, 90 kVp, 8–12 mA, field of view covering the entire maxillary sinus). Images were exported as DICOM files and analyzed in Planmeca Romexis 6.3 software.

- AI-Assisted Measurement Procedure

**Figure 1:**
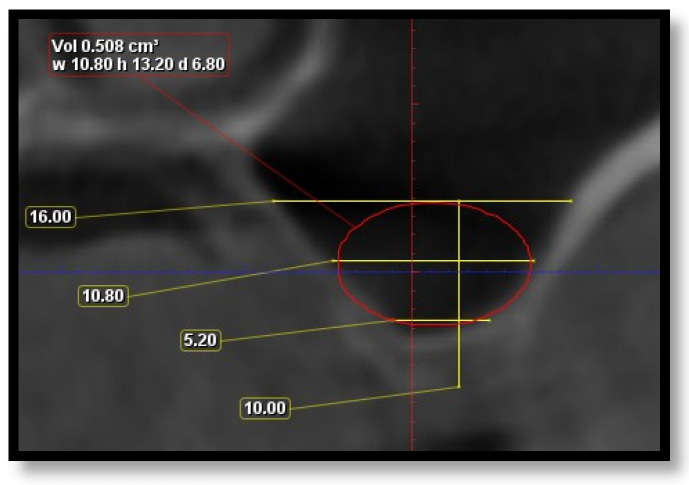
The area that AI tool will measure at sagittal view with its highest point at 10 mm implant height from the crest and extended to the buccal and palatal walls.

**Figure 2:**
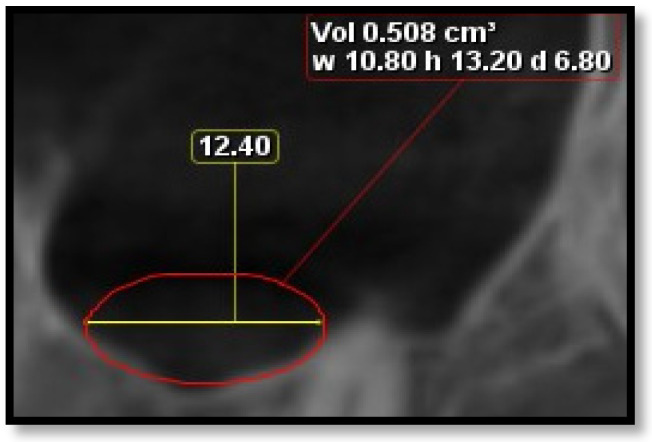
The area that AI tool extended mesially and distally to cover the bone augmenation area.

The AI-assisted volumetric estimation was performed using the volumetric analysis tool in the Planmeca Romexis software. The operator manually adjusted the elliptical region of interest (ROI) corresponding to the suggested augmentation envelope. In the sagittal view, the ROI was adjusted bucco-palatally and vertically according to the same reference points used for manual measurements, with the superior boundary corresponding to a projected implant height of 10 mm from the alveolar crest. In the panoramic view the ROI was extended mesially and distally to include the anticipated augmentation borders according to the planned implant sites. Figure 1 and Figure 2.

After setting the boundaries of the ROI, the software automatically calculated the volume of the augmentation in cubic centimeters (cm^3^). Hence, the procedure was a semi-automated operator-guided AI-assisted volumetric estimation rather than a fully autonomous sinus segmentation process. AI-assisted volumetric measurements were performed independently by two calibrated periodontists. Intra-examiner reproducibility was determined by repeating the measurements twice by each examiner with a two-week period between the sessions. For cases with more than one planned implant site, the total AI-derived augmentation volume was divided by the number of planned implant sites to derive an average augmentation volume per implant site.

- Standardization of Reference Points

To ensure comparability between manual and AI-assisted volumetric measurements, both methods were based on the same planned implant position and augmentation boundaries. The region of interest (ROI) used for AI-assisted volumetric estimation was defined using the same anatomical reference points employed during manual measurements, including the alveolar crest, projected implant height (10 mm), mesiodistal extent of the augmentation region, and buccopalatal dimensions of the planned grafting area. Consequently, both methods evaluated the same augmentation envelope and differed only in the approach used to calculate augmentation volume.

**Figure 3:**
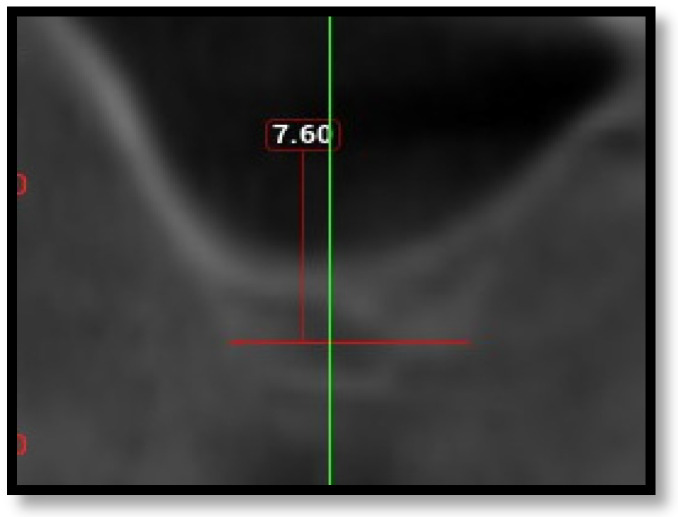
Bone Width, measured from buccal plate to palatal plate at the level of the bone crest (point A) in the sagittal view.

**Figure 4:**
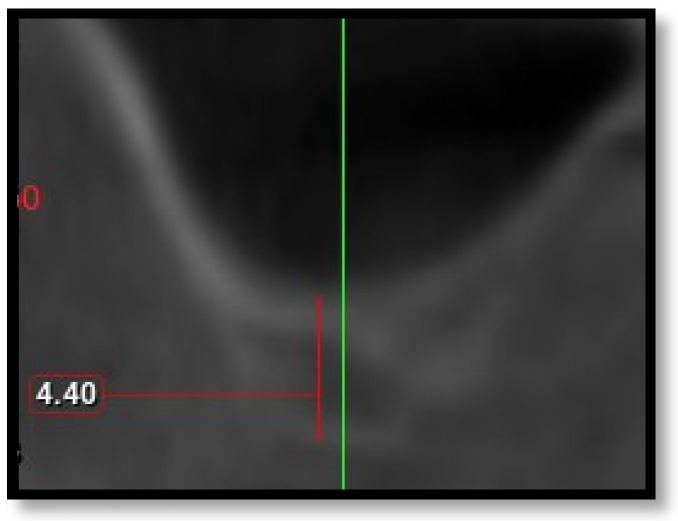
Residual alveolar bone height, Measured from the alveolar crest to the sinus floor (point B) in the sagittal view.

**Figure 5:**
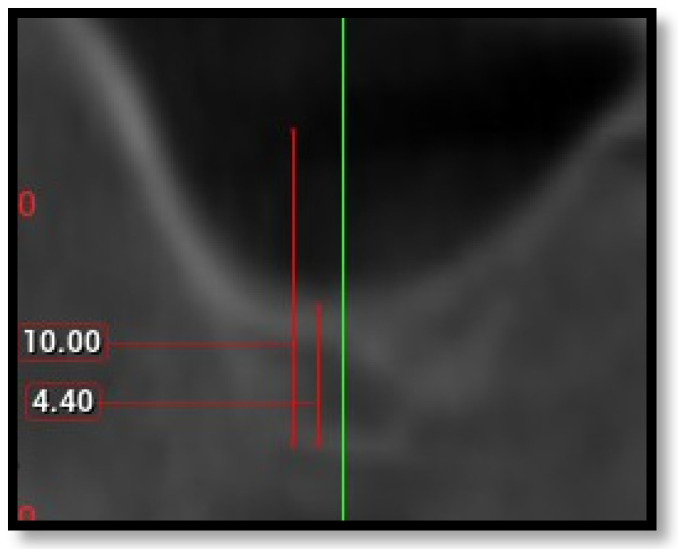
Maxillary sinus lift height, measured from sinus floor to the highest point of a projected 10 mm implant from the crest (point C) in the sagittal view.

**Figure 6:**
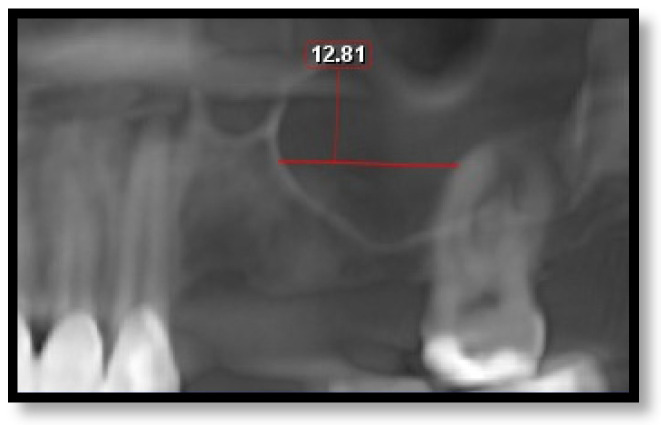
Maxillary sinus lift width, measured from mesial to distal sinus extension, 5 mm from the mid-crest (point D) in the panoramic view.

**Figure 7:**
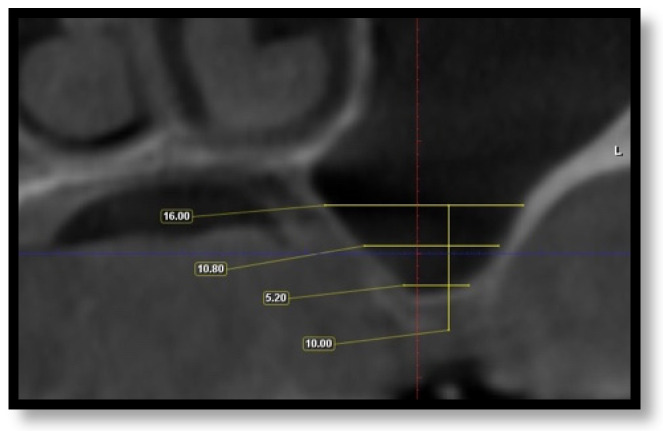
Average sinus depth (Point E) measured as an avergae from buccal–palatal at the apical (E1), mid (E2) and coronal (E3) part of the implant length.

- Manual Measurement Procedure
  Two calibrated periodontists independently performed manual measurements on sagittal and panoramic CBCT slices using the Planmeca Romexis ruler tool. Measurements followed standardized reference points as outlined below:
  - Bone width: Measured from buccal plate to palatal plate at the level of the bone crest (point A) in the sagittal view. Figure 3.
  - Residual alveolar bone height: Measured from the alveolar crest to the sinus floor (point B) in the sagittal view. Figure 4.
  - Maxillary sinus lift height: Measured from sinus floor to the highest point of a projected 10 mm implant from the crest (point C) in the sagittal view. Figure 5.
  - Maxillary sinus lift width: Measured from mesial to distal sinus extension, 5 mm from the mid-crest (point D) in the panoramic view. Figure 6.
  - Sinus depth (Apical): Measured buccal–palatal at the apical part of the implant length (point E1). Figure 7.
  - Sinus depth (Mid): Measured buccal–palatal at the mid-portion of implant length (point E2). Figure 7.
  - Sinus depth (Coronal): Measured buccal–palatal at the coronal part of implant length (point E3). Figure 7.
  - Average sinus depth: Mean of E1, E2, and E3 (E = [E1 + E2 + E3]/3). Figure 7.

Each examiner performed the measurements twice, with a two-week interval, to assess intra-examiner reliability. The mean of the repeated measurements from both examiners was used as the reference manual value for comparison with AI-assisted volumetric estimation. The average of the linear measurements was used to calculate the manual augmentation volume. For cases involving multiple planned implant sites within the same augmentation region, volumetric measurements were performed across the entire planned augmentation envelope. The resulting augmentation volume was subsequently normalized by the number of planned implant sites to obtain an average augmentation volume per implant site. Cases with sinus septa were not analyzed as a separate subgroup; however, scans were included provided that the planned augmentation region could be fully visualized and measured within the CBCT field of view.

Manual Sinus Volume Calculation

For manual volumetric estimation, the sinus was approximated as an ellipsoid-shaped cavity, and the volume was calculated using the following geometric formula:

- Sinus Volume (cm^3^) = 0.523 × (Width) × (Height) × (Depth)
- Where
  - Width (D) = Mesiodistal sinus dimension in the panoramic view.
  - Height (C) = Sinus lift height.
  - Depth (E) = Average bucco-palatal measurement (Average of E1, E2, E3).
- All linear measurements were taken in millimeters and converted to centimeters before volume calculations. The formula provides a close geometric approximation consistent with previous CBCT volumetric methods for paranasal sinuses.

Surgical Variables

The planned surgical approach was categorized based on the sinus lift approach required for each case by these categories:

- Internal sinus augmentation with simultaneous implant placement,
- External sinus augmentation followed by delayed implant placement.

Guided bone regeneration (GBR) planning was also recorded as either the case requires GBR or no GBR is needed.

Outcome Variables
  Primary Outcome

- Comparison between AI-assisted and manually calculated planned augmentation volume per implant site (cm^3^).

Secondary Outcomes

- Intra-examiner reliability of manual measurements.
- Inter-examiner reliability of manual measurements.
- Intra-examiner reliability of AI-assisted measurements.
- Inter-examiner reliability of AI-assisted measurements.
- Agreement between AI-assisted and manually calculated planned augmentation volumes.
- Relationship between age and volumetric measurements.
- Comparison of volumetric measurements according to sex.
- Comparison of volumetric measurements according to number of missing teeth.
- Distribution of clinical approaches and GBR utilization.

Statistical Analysis Plan

Descriptive statistics were reported as mean ± standard deviation (SD) for continuous variables and frequencies and percentages for categorical variables.

Pearson correlation analysis was used to evaluate:

- Age and manual planned augmentation volume.
- Age and AI-assisted planned augmentation volume.
- Age and radiographic measurements.

Independent sample *t*-tests were used to evaluate volumetric differences according to sex.

The comparison between AI-assisted and manually calculated planned augmentation volumes was performed using paired sample *t*-tests. Bland–Altman analysis was performed to assess agreement between AI-assisted and manual volumetric measurements by calculating mean bias and limits of agreement. Reliability and agreement analyses were performed using the Intraclass Correlation Coefficient (ICC) employing a two-way random-effects model with absolute agreement [ICC(2,1)]. The following analyses were performed:

- Manual A1 vs. A2 (intra-examiner reliability)
- Manual B1 vs. B2 (intra-examiner reliability)
- Manual A vs. B (inter-examiner reliability)
- AI A1 vs. A2 (intra-examiner reliability)
- AI B1 vs. B2 (intra-examiner reliability)
- AI A vs. B (inter-examiner reliability)
- AI-assisted versus manual measurements (agreement)

Statistical significance was set at *p* < 0.05. ChatGPT 5.6(OpenAI) was used as an auxiliary tool to assist with statistical formula verification and interpretation of statistical outputs. The statistical analyses and calculations were primarily performed using Microsoft Excel and independently reviewed by the investigators.

## 3. Results

### 3.1. Demographic Characteristics

A total of 60 patients were included in the study and it consisted of 30 males (50%) and 30 females (50%). There were 21 patients (35%) with a single missing tooth, 19 patients (32%) with two missing teeth, and 20 patients (33%) with three missing teeth. Table 1.

### 3.2. Radiographic Measurements

The mean bone width was 6.05 ± 0.41 mm, while the mean residual alveolar bone height was 4.12 ± 0.69 mm. The mean planned sinus augmentation dimensions demonstrated a sinus lift height of 5.92 ± 0.72 mm, a sinus lift width of 15.39 ± 1.77 mm, and an average sinus depth of 9.77 ± 0.70 mm (Table 2).

### 3.3. Comparison Between Manual and AI Volumetric Measurements

The mean manually calculated planned augmentation volume per implant site was 0.47 ± 0.11 cm^3^, whereas the mean AI-assisted planned augmentation volume per implant site was 0.52 ± 0.10 cm^3^. AI-assisted volumetric estimation produced significantly greater volume measurements than manual calculations (*p* < 0.001), indicating a small but consistent systematic increase in volume estimation by the AI-assisted method. Table 3.

### 3.4. Reliability and Agreement Analysis

Manual measurements demonstrated moderate reproducibility. The intra-examiner reliability of Periodontist A yielded an ICC value of 0.722, whereas Periodontist B demonstrated an ICC value of 0.701. Inter-examiner agreement between both periodontists demonstrated an ICC value of 0.649. AI-assisted volumetric measurements demonstrated excellent reproducibility. The intra-examiner ICC values were 0.917 and 0.924 for Periodontist A and Periodontist B, respectively. Inter-examiner agreement between both examiners was excellent, with an ICC value of 0.903. Agreement analysis between AI-assisted and manually calculated planned augmentation volumes demonstrated good agreement with an ICC value of 0.847. Table 4.

### 3.5. Bland–Altman Analysis

Bland–Altman analysis demonstrated a mean difference (bias) of 0.061 cm^3^, indicating that AI-assisted volumetric estimation consistently produced slightly greater volume measurements than manual calculations. All observed differences were positive, indicating a systematic tendency for AI-assisted measurements to produce larger volume estimates than manual calculations. The upper and lower limits of agreement were 0.094 cm^3^ and 0.028 cm^3^, respectively. These findings indicate a small but systematic overestimation by the AI-assisted method while maintaining acceptable agreement between both measurement techniques. Table 5.

### 3.6. Age Correlation Analysis

Pearson correlation analysis demonstrated no statistically significant correlation between age and any of the radiographic or volumetric measurements evaluated in the present study. Correlation coefficients ranged from −0.164 to 0.213, indicating negligible to weak associations between age and augmentation-related measurements. The strongest positive correlation was observed between age and sinus lift depth (E) (r = 0.213, *p* = 0.10), followed by age and manually calculated planned augmentation volume (r = 0.204, *p* = 0.12) and age and AI-assisted planned augmentation volume (r = 0.199, *p* = 0.13). However, none of these correlations reached statistical significance.

Similarly, bone width (A), sinus lift height (C), and sinus lift width (D) demonstrated weak positive correlations with age, whereas residual alveolar bone height (B) demonstrated a weak negative correlation (r = −0.164, *p* = 0.21). Overall, age was not found to significantly influence radiographic dimensions or planned augmentation volume measurements in the present study. Table 6.

### 3.7. Sex-Based Comparison

No statistically significant differences were observed between males and females for any of the radiographic dimensions or volumetric measurements evaluated in the present study. The mean bone width (A) was 6.14 ± 0.37 mm in females and 6.01 ± 0.43 mm in males (*p* = 0.107). Similarly, residual alveolar bone height (B) was 3.91 ± 0.65 mm in females and 4.18 ± 0.74 mm in males (*p* = 0.748). Sinus lift height (C), sinus lift width (D), and sinus lift depth (E) also demonstrated no statistically significant sex-related differences (*p* > 0.05).

Regarding volumetric measurements, the mean manually calculated planned augmentation volume per implant site was 0.44 ± 0.11 cm^3^ in females and 0.48 ± 0.10 cm^3^ in males (*p* = 0.645). Similarly, the mean AI-assisted planned augmentation volume per implant site was 0.49 ± 0.12 cm^3^ in females and 0.54 ± 0.09 cm^3^ in males (*p* = 0.687).

Although males demonstrated slightly greater radiographic dimensions and augmentation volumes than females, none of these differences reached statistical significance, indicating that sex was not a significant determinant of augmentation-related measurements in the present study. Table 7.

### 3.8. Analysis According to Number of Missing Teeth

After normalization of augmentation volume according to the number of planned implant sites, no statistically significant differences were observed between one-, two-, and three-tooth edentulous spans. The mean manually calculated augmentation volume per implant site ranged from 0.45 to 0.48 cm^3^, while AI-assisted measurements ranged from 0.51 to 0.56 cm^3^. Pairwise comparisons between groups demonstrated no statistically significant differences (*p* > 0.05). Table 8 and Table 9.

### 3.9. Surgical Approach and GBR Analysis

Internal sinus augmentation with simultaneous implant placement was performed in 25 cases (42%), whereas external sinus augmentation followed by delayed implant placement was performed in 35 cases (58%). The mean manually calculated augmentation volume per implant site was 0.43 cm^3^ in the internal augmentation group and 0.49 cm^3^ in the external augmentation group. Corresponding AI-assisted volumes were 0.50 cm^3^ and 0.56 cm^3^, respectively. Guided bone regeneration (GBR) was required in 25 cases (42%), whereas 35 cases (58%) did not require GBR. Table 10, Table 11 and Table 12.

## 4. Discussion

The aim of the present study was to evaluate the agreement, reliability of a semi-automated AI-assisted volumetric estimation tool compared with CBCT-derived manually calculated augmentation volumes for sinus augmentation planning. The principal finding was that AI-assisted volumetric estimation produced statistically significantly greater augmentation volume measurements than manual calculations, with mean values of 0.52 cm^3^ and 0.47 cm^3^, respectively (*p* < 0.001). Despite this systematic difference, agreement between both methods remained good (ICC = 0.847), suggesting that AI-assisted volumetric estimation may provide potentially useful augmentation volume estimates for clinical planning while improving measurement reproducibility [1].

CBCT imaging remains the imaging modality of choice for sinus augmentation planning because it allows three-dimensional assessment of residual bone dimensions, sinus anatomy, and augmentation requirements. In the present study, the mean residual alveolar bone height was 4.12 mm, indicating that many included sites represented moderate to severely atrophic posterior maxillae requiring augmentation procedures. The mean augmentation dimensions of 5.92 mm in height, 15.39 mm in width, and 9.77 mm in depth further emphasize the importance of accurate volumetric planning in implant dentistry and regenerative periodontology [2].

One of the main findings was the difference in reproducibility between manual and AI-assisted measurements. Manual measurements demonstrated moderate intra-examiner reliability (ICC = 0.722 and 0.701) and moderate inter-examiner reliability (ICC = 0.649). In contrast, AI-assisted measurements showed excellent intra-examiner reliability (ICC = 0.917 and 0.924) and excellent inter-examiner reliability (ICC = 0.903). These results show that AI-assisted volumetric estimation greatly reduces operator-dependent variability and improves standardization of measurements. The AI-assisted workflow, which employs operator-guided augmentation boundaries with automated volume calculation, reduces variability associated with repeated linear measurements and geometric calculations [9].

The intra-examiner reliability analysis demonstrated that AI-assisted measurements were considerably more reproducible than manually calculated measurements. These findings suggest that AI-assisted volumetric estimation may reduce operator-dependent variability and improve consistency during augmentation planning, particularly when repeated measurements are required.

Analysis of agreement between AI-assisted and manually calculated augmentation volumes showed good agreement (ICC = 0.847). This degree of agreement is of clinical importance because the two methods are fundamentally different. Manual volumetric estimation relies on geometric approximation using representative linear measurements, and the AI-assisted workflow quantifies the augmentation envelope as a volumetric region of interest. Hence, perfect agreement is neither expected nor required. The degree of agreement observed suggests that AI-assisted volumetric estimation may provide clinically acceptable augmentation volume measurements despite methodological differences from manual calculations.

An important consideration when interpreting the present findings is that the evaluated AI system was a semi-automated operator-guided volumetric estimation tool rather than a fully autonomous segmentation algorithm. Unlike fully automated deep-learning segmentation systems, the present workflow required the operator to define the augmentation boundaries before automatic volume calculation. This approach may reduce segmentation errors associated with anatomical variability while maintaining clinician oversight of the region of interest. However, fully automated segmentation systems have the potential to further reduce operator involvement and improve efficiency. Future studies should directly compare semi-automated volumetric estimation with fully autonomous three-dimensional segmentation approaches to determine whether additional improvements in accuracy and reproducibility can be achieved.

The volumetric measurements in the present study were standardized using a projected implant height of 10 mm from the alveolar crest. This reference was selected because a 10 mm implant represents a commonly used implant length in posterior maxillary rehabilitation and provides a clinically relevant benchmark for augmentation planning. By standardizing the superior boundary of the augmentation envelope, measurements could be compared consistently between patients and between manual and AI-assisted methods. Consequently, the calculated augmentation volume reflects the amount of grafting required to facilitate placement of a clinically representative implant length.

The Bland–Altman analysis revealed a mean bias of 0.061 cm^3^, suggesting that the AI-assisted measurements yielded consistently larger augmentation volumes than the manual calculations. The limits of agreement ranged from 0.028 cm^3^ to 0.094 cm^3^. This shows a small but systematic bias of the AI-assisted method towards overestimation of volumes. Such a difference may translate clinically to the ability of the volumetric tool to encompass a wider augmentation envelope than geometric calculations derived from representative linear dimensions. Despite this systematic difference, the limits of agreement were relatively narrow, suggesting that the AI-assisted method may have potential utility in augmentation planning, although clinical applicability should be confirmed in prospective clinical studies [20].

Although AI-assisted volumetric estimation produced statistically greater augmentation volumes than manual calculations, the observed mean difference was relatively small (0.061 cm^3^). From a clinical perspective, such a difference is unlikely to substantially alter treatment planning decisions or graft material selection in most cases. However, the tendency of AI-assisted measurements to produce slightly larger volume estimates may provide a small safety margin during augmentation planning and could potentially reduce the risk of underestimating graft requirements. More importantly, the excellent reproducibility demonstrated by the AI-assisted workflow suggests that improved standardization may be clinically more valuable than the small absolute difference observed between the two measurement methods.

The present findings support the growing role of AI-assisted image analysis in digital implantology and CBCT-based treatment planning. Previous investigations utilizing deep-learning and segmentation algorithms demonstrated promising accuracy and reproducibility in maxillary sinus analysis. Although the current study evaluated a semi-automated operator-guided tool rather than a fully autonomous segmentation system, the excellent reproducibility and good agreement observed support the integration of AI-assisted volumetric estimation into routine preoperative planning workflows [20].

No statistically significant correlations were identified between age and any radiographic or volumetric variable evaluated in the present study. Correlation coefficients ranged from −0.164 to 0.213 and all *p*-values exceeded 0.05. These findings indicate that chronological age alone does not significantly influence augmentation-related dimensions or volumetric requirements in patients undergoing implant treatment.

Similarly, no statistically significant sex-related differences were observed for radiographic dimensions or volumetric measurements. Although males demonstrated slightly larger measurements than females, none of these differences reached statistical significance. These findings suggest that sex has limited influence on augmentation-related dimensions in the present patient population and support previous reports demonstrating substantial individual anatomical variability independent of sex [11].

After normalization of augmentation volume according to the number of planned implant sites, no statistically significant differences were observed between one-, two-, and three-tooth edentulous spans. This finding differs from previous observations in which wider edentulous spans were associated with greater total augmentation volumes. However, because the present study evaluated augmentation volume per implant site rather than total augmentation volume per patient, the absence of significant differences suggests that augmentation requirements remained relatively consistent at the implant-site level regardless of the extent of tooth loss [13].

The distribution of clinical approaches further reflected the anatomical complexity of the included cases. External sinus augmentation followed by delayed implant placement was indicated in 58% of cases, whereas internal sinus augmentation with simultaneous implant placement was indicated in 42% of cases. Sites requiring external augmentation demonstrated larger augmentation volume requirements than those managed with internal augmentation. This finding is consistent with established treatment concepts in which more severe atrophy and larger augmentation requirements favor a staged lateral-window approach [16].

The present study was intentionally focused on volumetric estimation and measurement reproducibility rather than anatomical risk factors associated with sinus augmentation. Therefore, variables such as sinus septa, membrane thickness, and sinus pathology were beyond the scope of the current investigation and warrant evaluation in future studies.

### 4.1. Limitations

There are some limitations to take into account. Firstly, the sample size was limited to 60 patients, which may reduce the statistical power for subgroup analyses. Second, manual volumetric estimation was based on a geometric ellipsoid approximation, not a full 3D segmentation, which could introduce approximation error. Thirdly, the evaluation was limited to a single CBCT system and a single AI-assisted software platform (Planmeca Romexis 6.3 software), which limits the generalizability to other imaging systems and AI tools. Fourth, the present study used an operator-guided AI-assisted workflow and therefore was not a fully autonomous segmentation system. Finally, anatomical variables like sinus septa, membrane thickening, and sinus pathology were not stratified independently, which might affect the performance of the volumetric estimation [20].

### 4.2. Future Directions

Future studies should include larger multicenter datasets acquired from multiple CBCT systems and AI platforms to improve external validity and generalizability. Additional investigations should evaluate AI-assisted volumetric estimation in anatomically complex sinuses with septa, mucosal thickening, pathological changes, and advanced pneumatization patterns. Comparisons with fully automated three-dimensional segmentation systems should also be performed to determine whether complete volumetric rendering provides superior accuracy compared with operator-guided volumetric estimation. Furthermore, longitudinal clinical studies are needed to determine whether AI-assisted volumetric planning improves graft stability, implant survival, surgical efficiency, and long-term treatment outcomes [20].

## 5. Conclusions

Within the limitations of this study, AI-assisted volumetric estimation produced slightly greater planned augmentation volumes than manually calculated CBCT-derived measurements, although agreement between both methods remained good. Manual measurements demonstrated moderate intra- and inter-examiner reliability, whereas AI-assisted measurements demonstrated excellent reproducibility and examiner agreement. No significant associations were observed between augmentation volume and age, sex, or number of missing teeth after normalization per implant site. The results indicate that AI-assisted volumetric estimation may serve as a reliable adjunctive tool for sinus augmentation planning by improving reproducibility and reducing operator-dependent variability. Clinician supervision remains essential, and further validation on larger heterogeneous datasets and fully automated segmentation approaches is recommended before AI-assisted volumetric estimation can be considered interchangeable with expert assessment.

## Figures and Tables

**Table 1:** Demographic characteristics and distribution of missing teeth.

**Variable**	***n (%)***
Female	30 (50%)
Male	30 (50%)
**Missing Teeth**	***n (%)***
1 missing tooth	21 (35%)
2 missing teeth	19 (32%)
3 missing teeth	20 (33%)

**Table 2:** Radiographic dimensional measurements.

Variable	Mean ± SD (mm)
Bone width	6.05 ± 0.41
Residual alveolar bone height	4.12 ± 0.69
Maxillary sinus lift height	5.92 ± 0.72
Maxillary sinus lift width	15.39 ± 1.77
Maxillary sinus lift depth	9.77 ± 0.70

**Table 3:** Comparison between manually calculated and AI-assisted planned augmentation volumes.

Measurement Method	Mean ± SD (mm)	*p*-Value
Manual volumetric estimation	0.47 ± 0.11	<0.001
AI-assisted volumetric estimation	0.52 ± 0.10	

**Table 4:** Reliability and agreement analysis using ICC.

Reliability Analysis	ICC Value	Interpretation
Periodontist A intra-examiner reliability (Manual)	0.722	Moderate
Periodontist B intra-examiner reliability (Manual)	0.701	Moderate
Inter-examiner reliability (Manual A vs. B)	0.649	Moderate
Periodontist A intra-examiner reliability (AI)	0.917	Excellent
Periodontist B intra-examiner reliability (AI)	0.924	Excellent
Inter-examiner reliability (AI A vs. B)	0.903	Excellent
AI vs. Manual agreement	0.847	Good

**Table 5:** Bland–Altman analysis.

Parameter	Value
Mean difference (AI − Manual)	0.061 cm^3^
SD of differences	0.017
Upper limit of agreement	0.094
Lower limit of agreement	0.028

**Table 6:** Pearson correlation analysis.

Variable	Pearson r	*p*-Value
Bone Width (A)	0.067	0.61
Residual Alveolar Bone Height (B)	−0.164	0.21
Sinus Lift Height (C)	0.158	0.23
Sinus Lift Width (D)	0.107	0.42
Sinus Lift Depth (E)	0.213	0.10
Manual Planned Augmentation Volume	0.204	0.12
AI-Assisted Planned Augmentation Volume	0.199	0.13

**Table 7:** Comparison of radiographic dimensions and volumetric measurements according to sex.

Variable	Female Mean ± SD	Male Mean ± SD
Bone Width (A)	6.14 ± 0.37	6.01 ± 0.43
Residual Bone Height (B)	3.91 ± 0.65	4.18 ± 0.74
Sinus Lift Height (C)	6.09 ± 0.65	5.82 ± 0.79
Sinus Lift Width (D)	15.21 ± 1.92	16.00 ± 1.61
Sinus Lift Depth (E)	9.74 ± 0.75	9.78 ± 0.67
Manual Volume (cm^3^)	0.44 ± 0.11	0.48 ± 0.10
AI Volume (cm^3^)	0.49 ± 0.12	0.54 ± 0.09

**Table 8:** Comparison of volumetric measurements according to number of missing teeth.

Missing Teeth	Manual Mean ± SD	AI Mean ± SD
1 missing tooth	0.45 ± 0.16	0.51 ± 0.16
2 missing teeth	0.48 ± 0.07	0.56 ± 0.07
3 missing teeth	0.48 ± 0.04	0.53 ± 0.05

**Table 9:** Pairwise comparison between missing-teeth groups.

Comparison	Manual *p*-Value	AI *p*-Value
1 missing tooth vs. 2 missing teeth	0.929	0.829
1 missing tooth vs. 3 missing teeth	0.599	0.452
2 missing teeth vs. 3 missing teeth	0.391	0.317

**Table 10:** Distribution of surgical approaches.

Surgical Approach	n (%)
Internal with implant	25 (42%)
External followed by implant later	35 (58%)

**Table 11:** Mean planned augmentation volume according to clinical approach.

Approach	Manual Volume (cm^3^)	AI Volume (cm^3^)
Internal with implant	0.43	0.50
External followed by implant later	0.49	0.56

**Table 12:** Guided bone regeneration analysis.

Procedure	n (%)
GBR	25 (42%)
No GBR	35 (58%)
Procedure	n (%)

## Data Availability

The data supporting the findings of this study are available from the corresponding author upon reasonable request. The data are not publicly available because they contain information derived from clinical records and radiographic images that are subject to privacy and ethical restrictions.

## Abbreviations

The following abbreviations are used in this manuscript:

AI	Artificial Intelligence
CBCT	Cone-Beam Computed Tomography
CNN	Convolutional Neural Network
GBR	Guided Bone Regeneration
ICC	Intraclass Correlation Coefficient
MSV	Maxillary Sinus Volume
REC	Research Ethics Committee
ROI	Region of Interest
SD	Standard Deviation
SFA	Sinus Floor Augmentation
SFE	Sinus Floor Elevation
U-Net	U-shaped Convolutional Neural Network Architecture
DICOM	Digital Imaging and Communications in Medicine
FOV	Field of View
LOA	Limits of Agreement
PMCID	PubMed Central Identifier
PMID	PubMed Identifier
cm^3^	Cubic Centimeter
mm	Millimeter
kVp	Kilovolt Peak
mA	Milliampere
A	Bone Width
B	Residual Alveolar Bone Height
C	Sinus Lift Height
D	Sinus Lift Width
E	Average Sinus Lift Depth
E1	Apical Sinus Depth Measurement
E2	Mid Sinus Depth Measurement
E3	Coronal Sinus Depth Measurement
